# Supplementary material for: End-to-end evaluation of pipelines for metagenome-assembled genomes reveals hidden performance gaps
Source: bioRxiv. 2026 Apr 9:2026.04.06.712906. Preprint. [Version 1] doi: 10.64898/2026.04.06.712906 (PMC13081952; doi:10.64898/2026.04.06.712906)
Supplement: 1 [file NIHPP2026.04.06.712906V1-supplement-1.pdf]

bioRxiv preprint doi: https://doi.org/10.64898/2026.04.06.712906; this version posted April 9, 2026. The copyright holder for this preprint (which was not certified by peer review) is the author/funder, who has granted bioRxiv a license to display the preprint in perpetuity. It is made available under aCC-BY 4.0 International license.

Supplementary figures

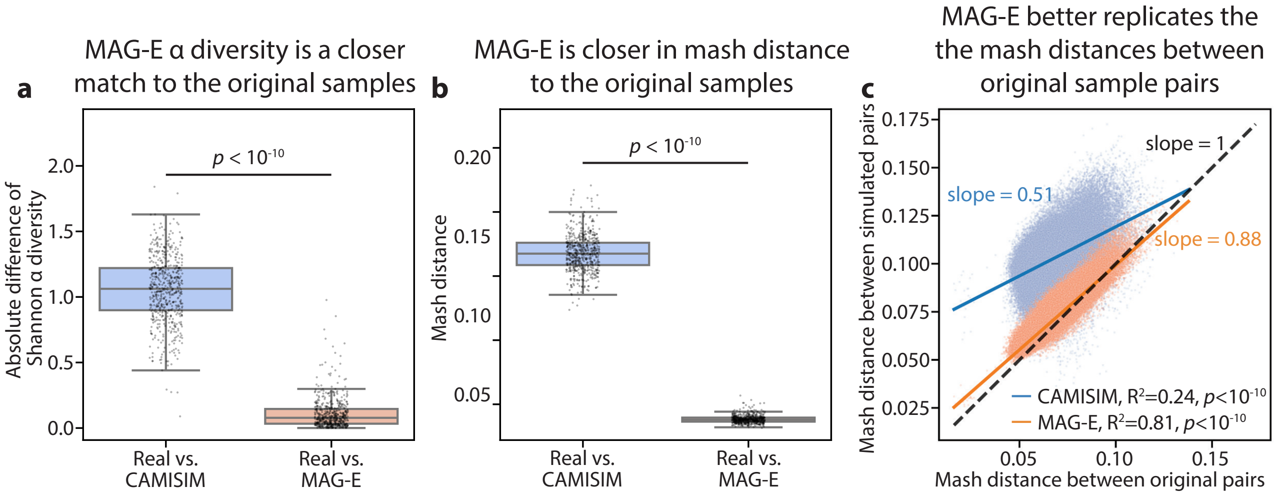

Supplementary Figure 1. a, Difference in Shannon α diversity between simulated and original metagenomes for 575 human gut metagenomes, comparing between CAMISIM and MAG-E. **b-c,** Same as **Fig. 1e,f**, respectively, using Mash distance instead of β diversity. Box, IQR; line, median; whiskers, 1.5xIQR; *p*, Wilcoxon signed-rank test (a,b) or Student's t-test (c).

bioRxiv preprint doi: https://doi.org/10.64898/2026.04.06.712906; this version posted April 9, 2026. The copyright holder for this preprint (which was not certified by peer review) is the author/funder, who has granted bioRxiv a license to display the preprint in perpetuity. It is made available under aCC-BY 4.0 International license.

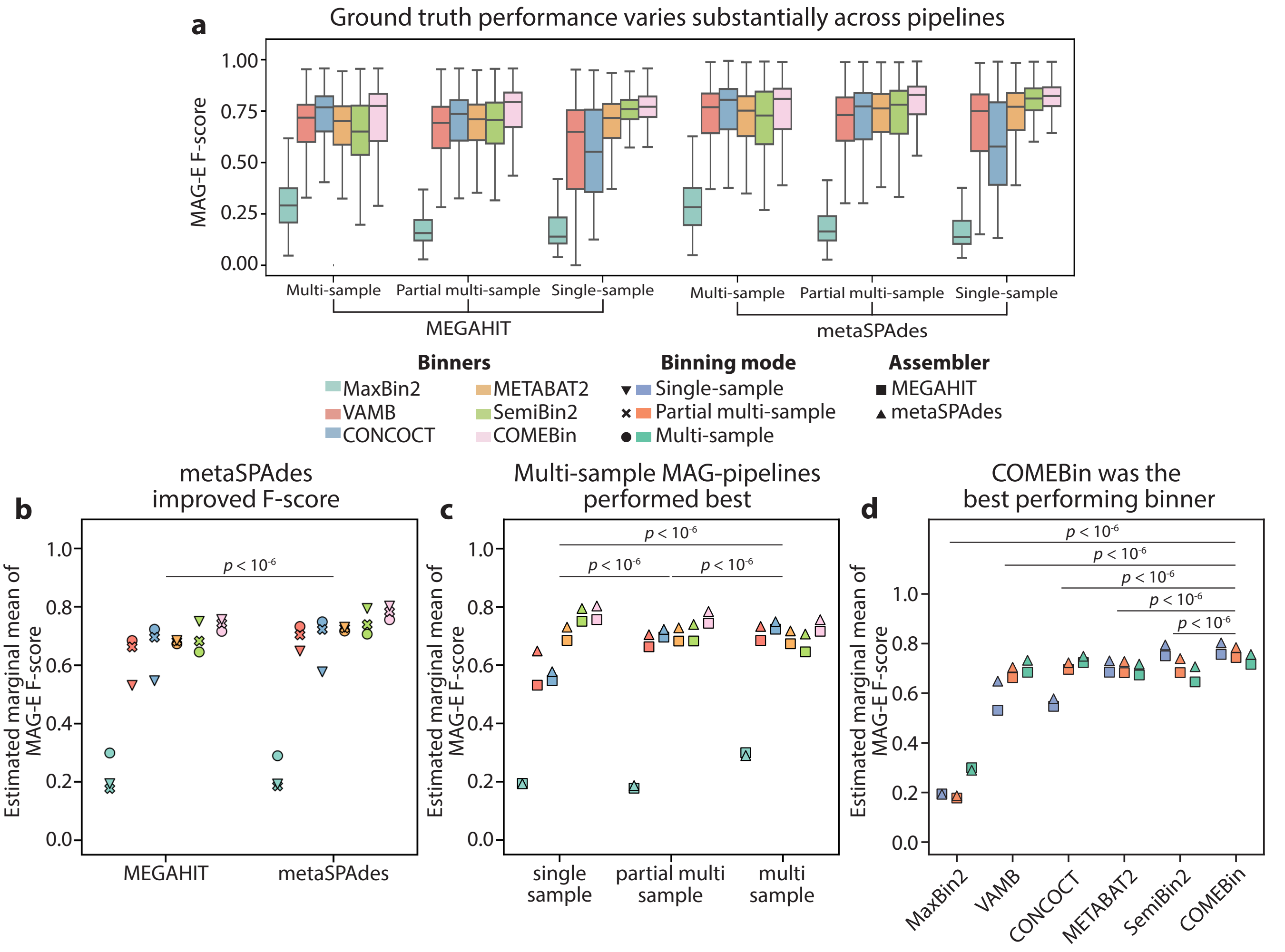

Supplementary Figure 2 | The MAG pipeline of metaSPAdes followed by multi-sample COMEBin outperforms alternatives based on F-scores. a-d, Same as **Fig. 2a,c,e,g**, respectively, showing F-score instead of recall.

bioRxiv preprint doi: https://doi.org/10.64898/2026.04.06.712906; this version posted April 9, 2026. The copyright holder for this preprint (which was not certified by peer review) is the author/funder, who has granted bioRxiv a license to display the preprint in perpetuity. It is made available under aCC-BY 4.0 International license.

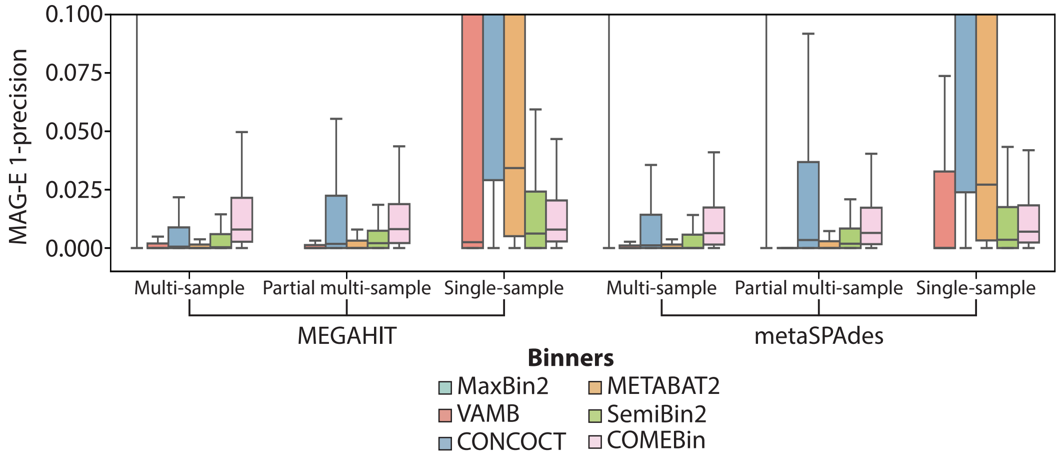

Supplementary Figure 3. 1-Precision of MAG pipelines over the [0, 0.1] range. Box, IQR; line, median; whiskers, 1.5xIQR.

bioRxiv preprint doi: https://doi.org/10.64898/2026.04.06.712906; this version posted April 9, 2026. The copyright holder for this preprint (which was not certified by peer review) is the author/funder, who has granted bioRxiv a license to display the preprint in perpetuity. It is made available under aCC-BY 4.0 International license.

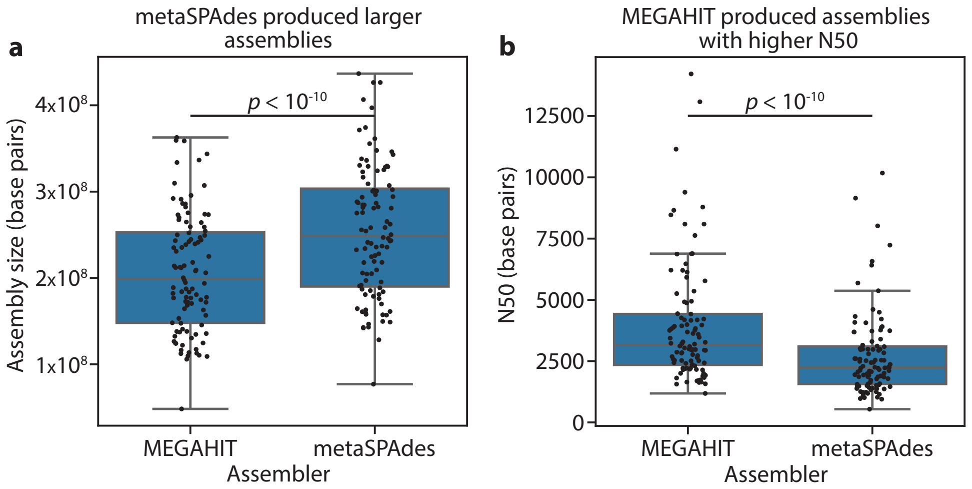

Supplementary Figure 4 | Assembly statistics for metaSPAdes vs MEGAHIT on the 100 samples evaluated with MAG-E. N50 (**a**) and assembly size (**b**; measured as the total number of basepairs) of each MEGAHIT and metaSPAdes assembly. Boxplot, IQR; line, median; whiskers, 1.5xIQR; *p*, Wilcoxon signed-rank test.

bioRxiv preprint doi: https://doi.org/10.64898/2026.04.06.712906; this version posted April 9, 2026. The copyright holder for this preprint (which was not certified by peer review) is the author/funder, who has granted bioRxiv a license to display the preprint in perpetuity. It is made available under aCC-BY 4.0 International license.

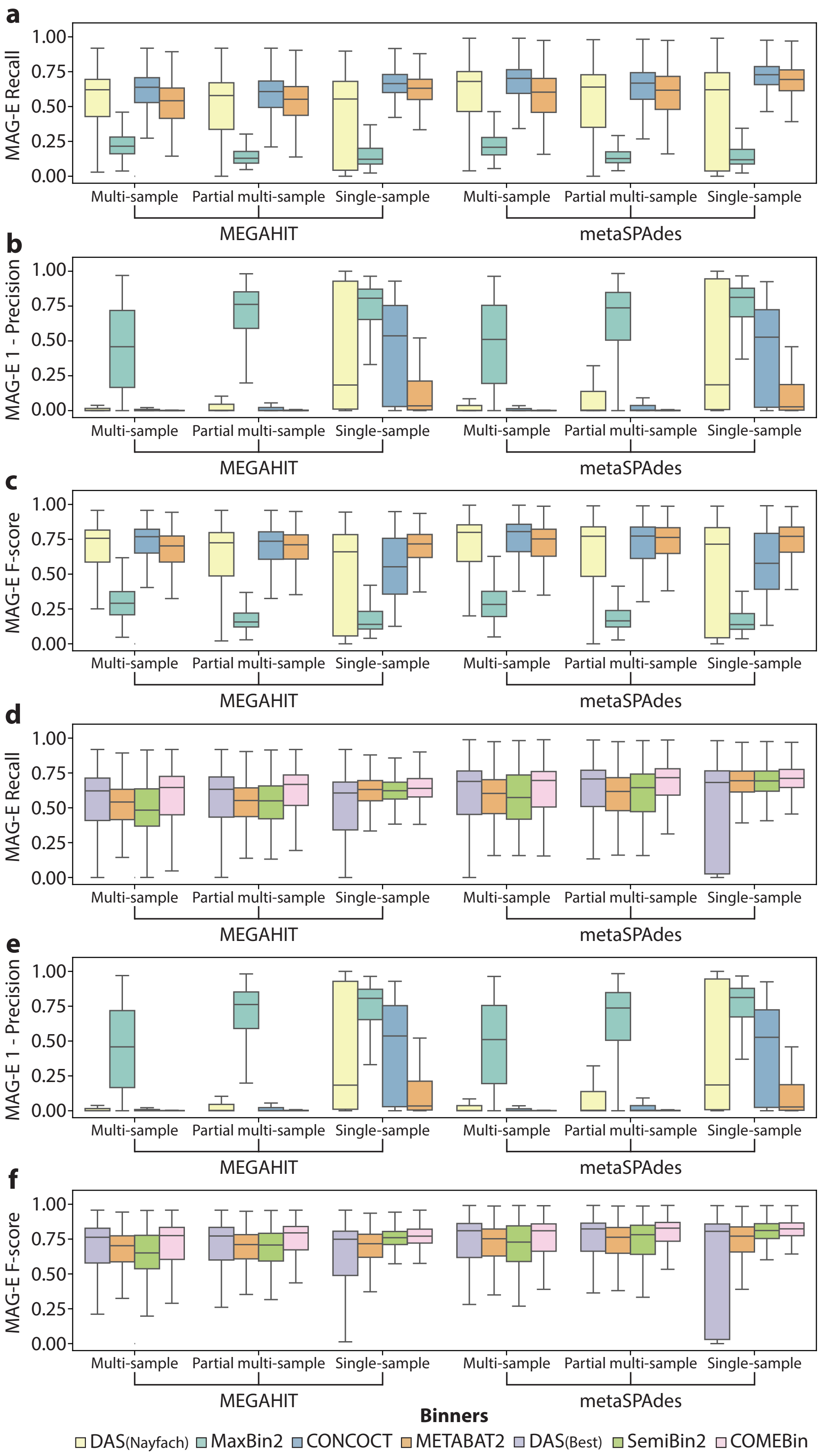

Supplementary Figure 5 | MAG-E recall, 1-precision and F_score for DAS Tool and associated binners. a-c, Boxplots showing MAG-E recall (a), 1-Precision (b) and F-score (c) of the recoverable genomes across all samples for DAS(Nayfach) and the binners it combines. **d-f**, same as (a-c) respectively, but for DAS(Best) and the binners it combines. Box, IQR; line, median; whiskers, 1.5xIQR.

bioRxiv preprint doi: https://doi.org/10.64898/2026.04.06.712906; this version posted April 9, 2026. The copyright holder for this preprint (which was not certified by peer review) is the author/funder, who has granted bioRxiv a license to display the preprint in perpetuity. It is made available under aCC-BY 4.0 International license.

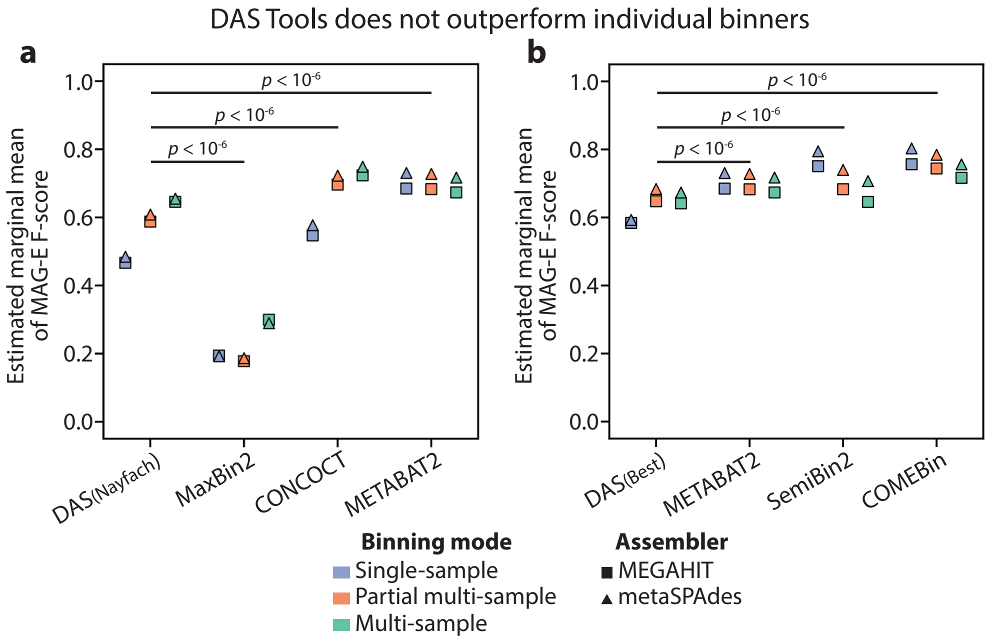

Supplementary Figure 6 | Applying DAS tools does not improve performance over individual binners. a,b, Same as **Fig. 3a,c**, respectively, showing F-score instead of recall. Two-sided Wald z-test, $p<10^{-6}$, Tukey HSD adjusted.

bioRxiv preprint doi: https://doi.org/10.64898/2026.04.06.712906; this version posted April 9, 2026. The copyright holder for this preprint (which was not certified by peer review) is the author/funder, who has granted bioRxiv a license to display the preprint in perpetuity. It is made available under aCC-BY 4.0 International license.

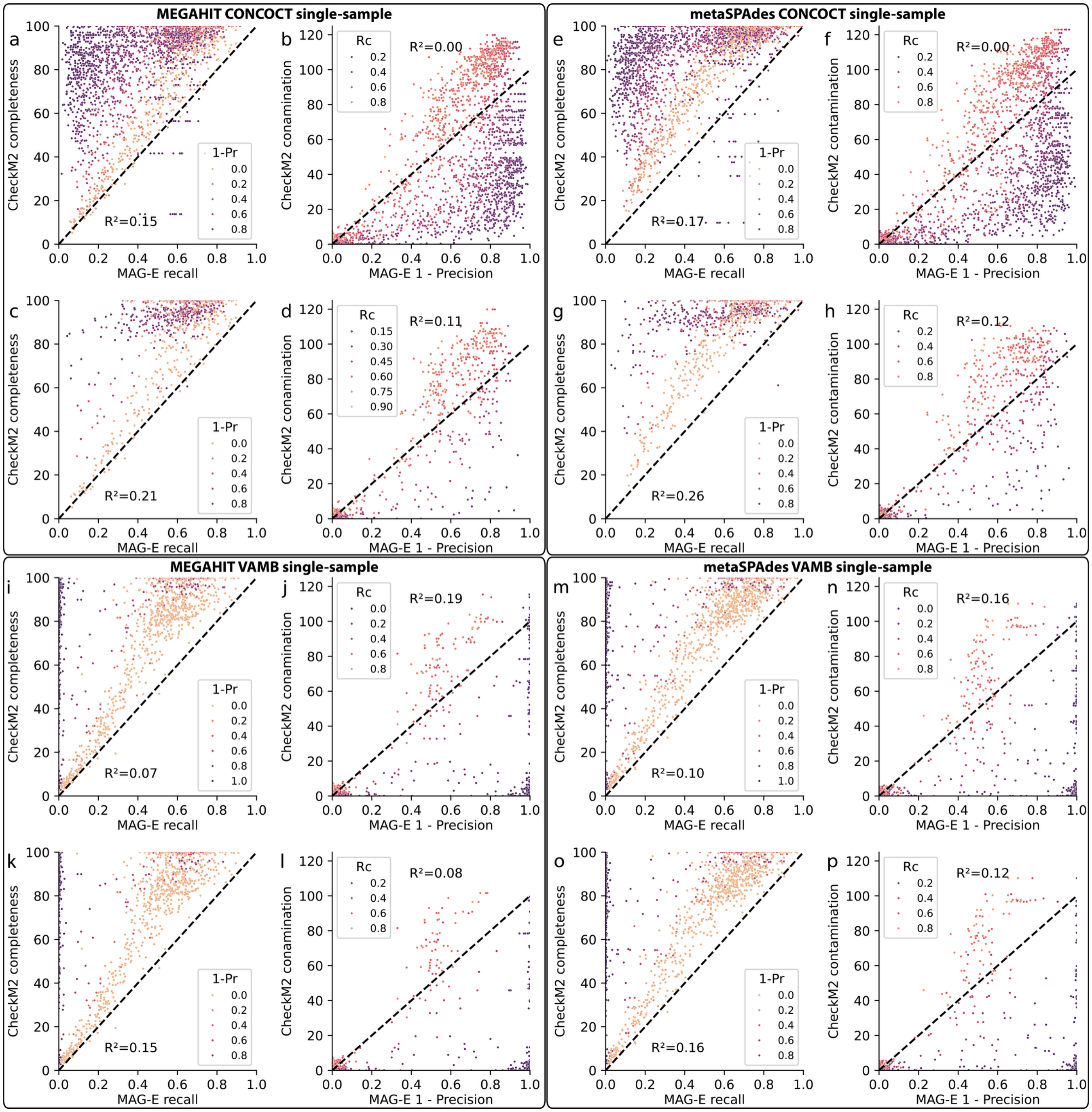

Supplementary Figure 7 | Evaluation of CheckM2 completeness and contamination versus ground-truth, per-genome MAG-E evaluation for CONCOCT and VAMB in single-sample mode. a-p, Same as **Fig. 5c,d,g,h** for CONCOCT after MEGAHIT (a-d) or metaSPAdes (e-h) assembly, or VAMB following MEGAHIT (i-l) or metaSPAdes (m-p) assembly. Rc, recall; 1-Pr, 1-precision; black dashed line, y=100x, indicating perfect correspondence between checkM2 and MAG-E; R^2, marginal explained variance from a linear mixed model of ground truth from checkM2 predictions (**Methods**).

bioRxiv preprint doi: https://doi.org/10.64898/2026.04.06.712906; this version posted April 9, 2026. The copyright holder for this preprint (which was not certified by peer review) is the author/funder, who has granted bioRxiv a license to display the preprint in perpetuity. It is made available under aCC-BY 4.0 International license.

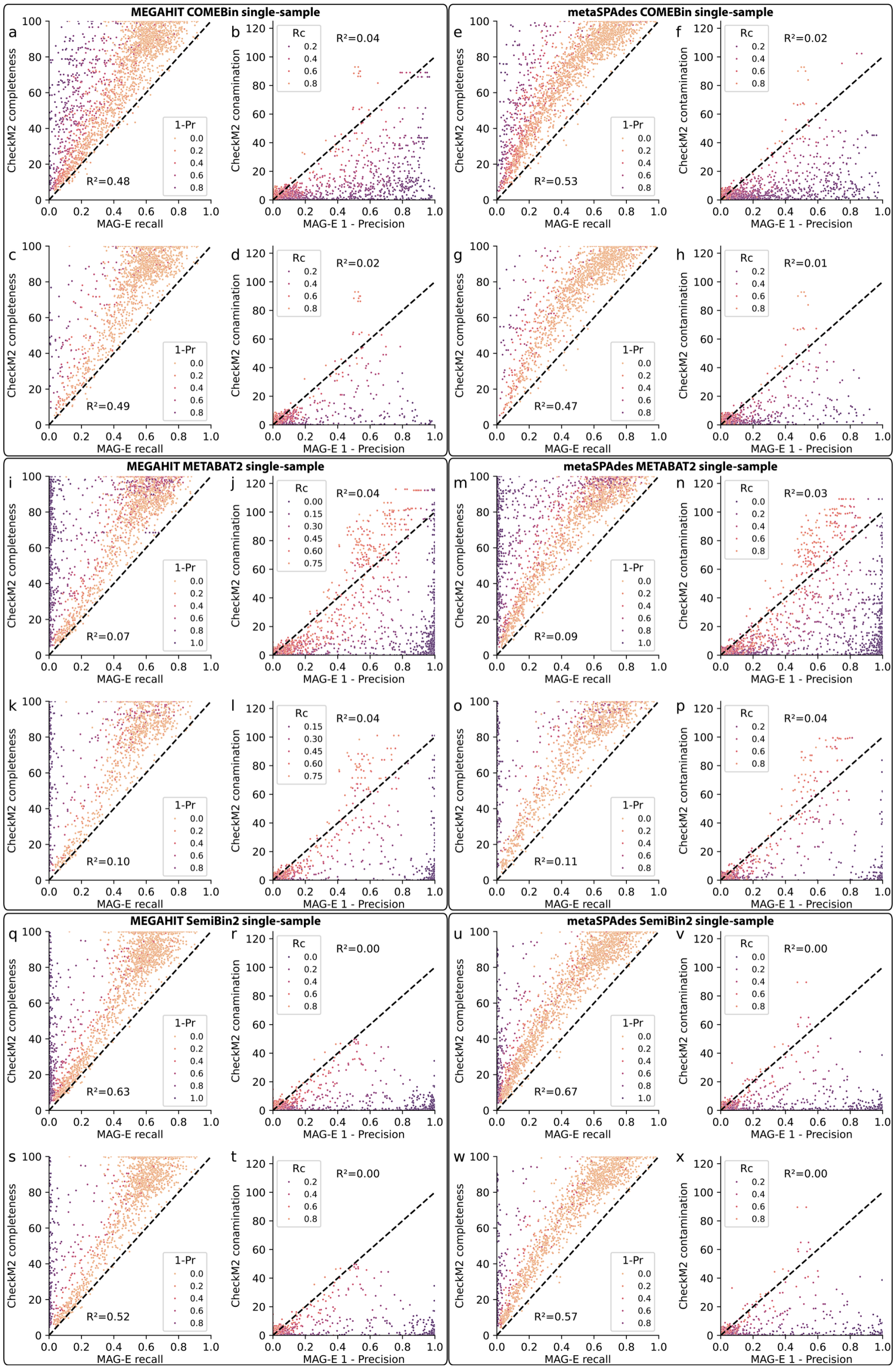

Supplementary Figure 8 | Evaluation of CheckM2 completeness and contamination versus ground-truth, per-genome MAG-E evaluation for COMEBin, METABAT2, and SemiBin2 in single-sample mode. a-x, Same as **Fig. 5c,d,g,h** for COMEBin after MEGAHIT (a-d) or metaSPAdes (e-h) assembly, METABAT2 binning after MEGAHIT (i-l) or metaSPAdes (m-p) assembly, or SemiBin2 following MEGAHIT (q-t) or metaSPAdes (u-x) assembly. Rc, recall; 1-Pr, 1-precision; black dashed line, y=100x, indicating perfect correspondence between checkM2 and MAG-E; R^2, marginal explained variance from a linear mixed model of ground truth from checkM2 predictions (**Methods**).

bioRxiv preprint doi: https://doi.org/10.64898/2026.04.06.712906; this version posted April 9, 2026. The copyright holder for this preprint (which was not certified by peer review) is the author/funder, who has granted bioRxiv a license to display the preprint in perpetuity. It is made available under aCC-BY 4.0 International license.

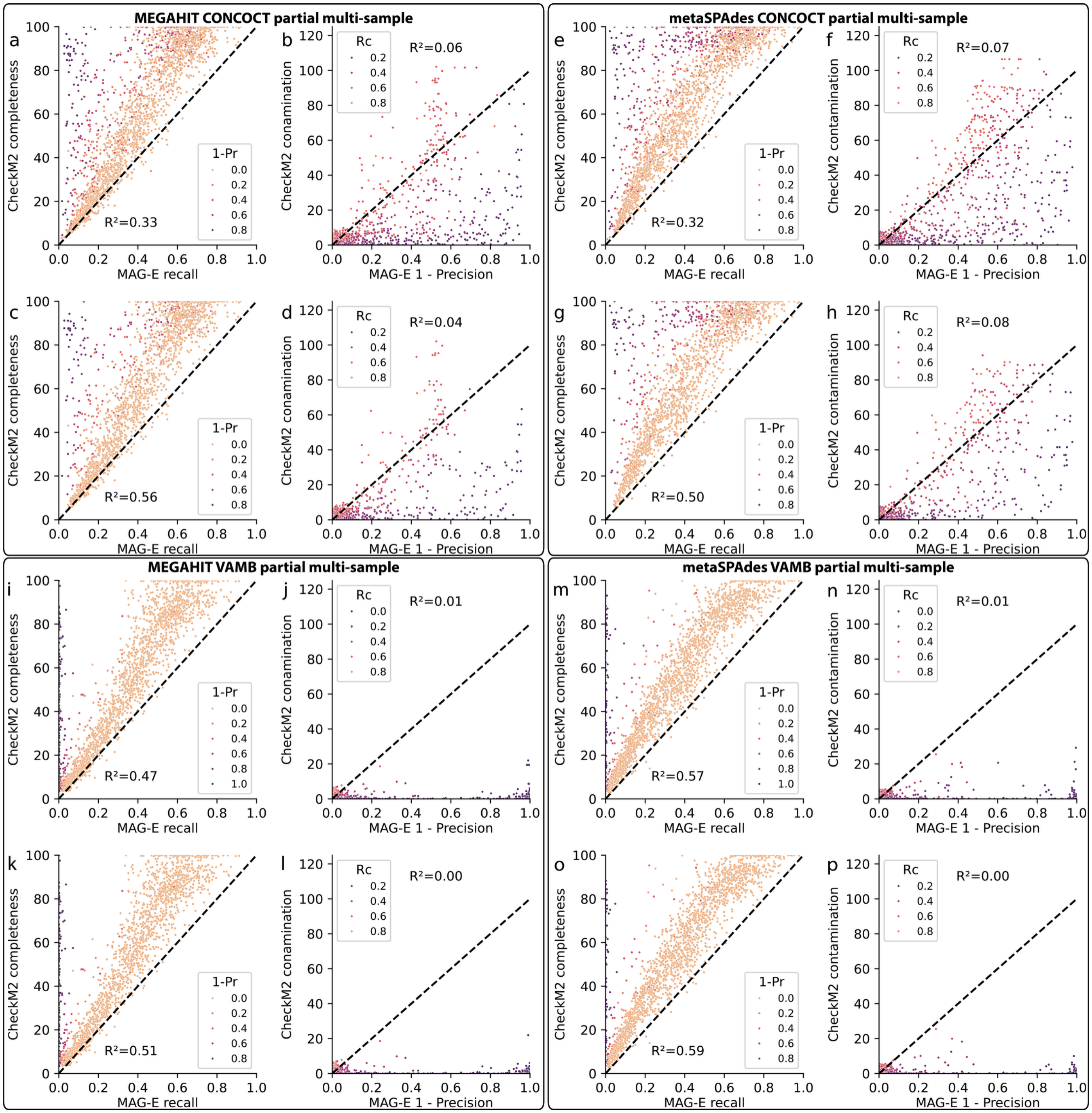

Supplementary Figure 9 | Evaluation of CheckM2 completeness and contamination versus ground-truth, per-genome MAG-E evaluation for CONCOCT and VAMB in partial multi-sample mode. Same as **Fig. S7** for partial multi-sample mode.

bioRxiv preprint doi: https://doi.org/10.64898/2026.04.06.712906; this version posted April 9, 2026. The copyright holder for this preprint (which was not certified by peer review) is the author/funder, who has granted bioRxiv a license to display the preprint in perpetuity. It is made available under aCC-BY 4.0 International license.

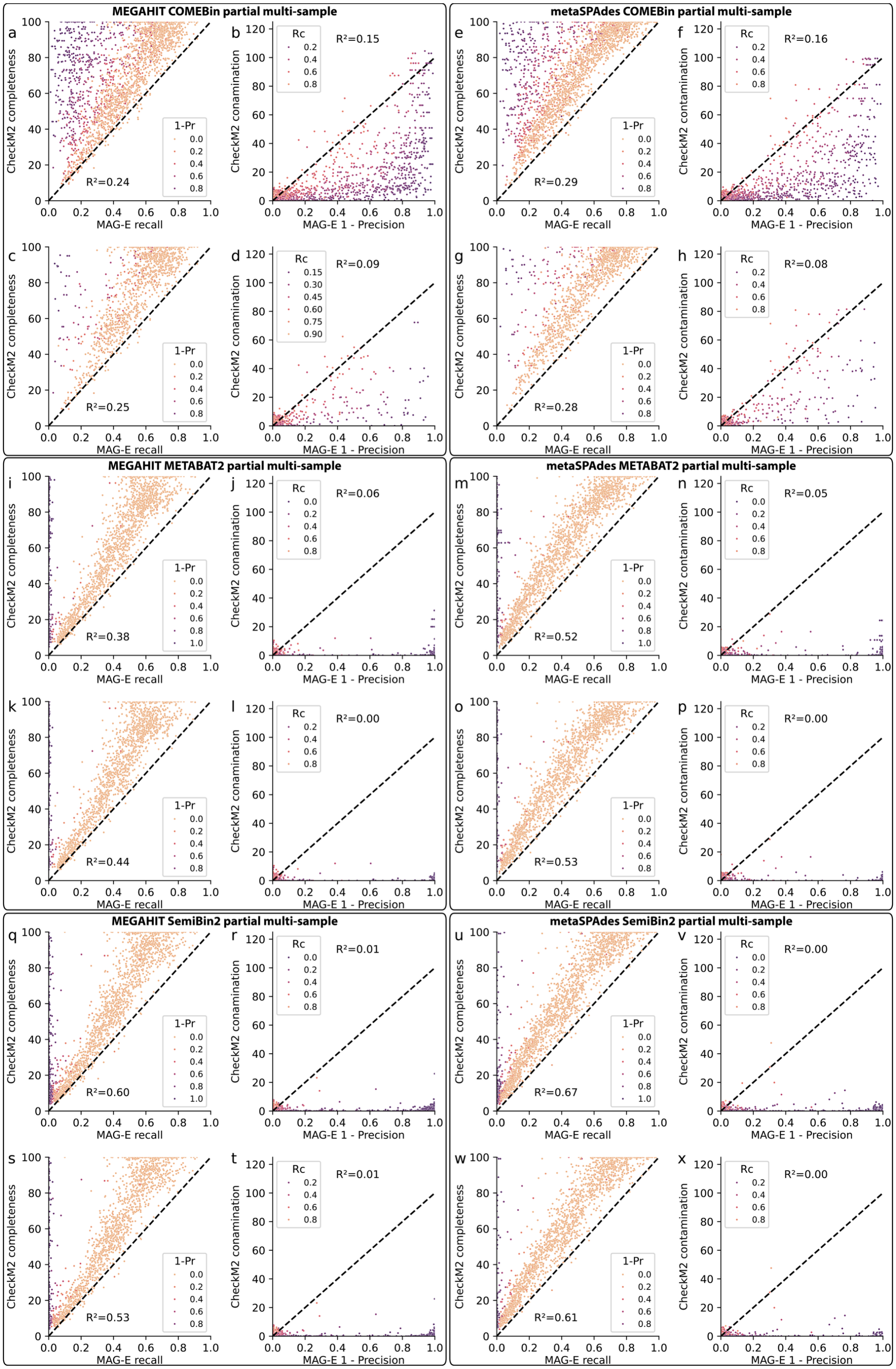

Supplementary Figure 10 | Evaluation of CheckM2 completeness and contamination versus ground-truth, per-genome MAG-E evaluation for COMEBin, METABAT2, and SemiBin2 in partial multi-sample mode. Same as **Fig. S8** for partial multi-sample mode.

bioRxiv preprint doi: https://doi.org/10.64898/2026.04.06.712906; this version posted April 9, 2026. The copyright holder for this preprint (which was not certified by peer review) is the author/funder, who has granted bioRxiv a license to display the preprint in perpetuity. It is made available under aCC-BY 4.0 International license.

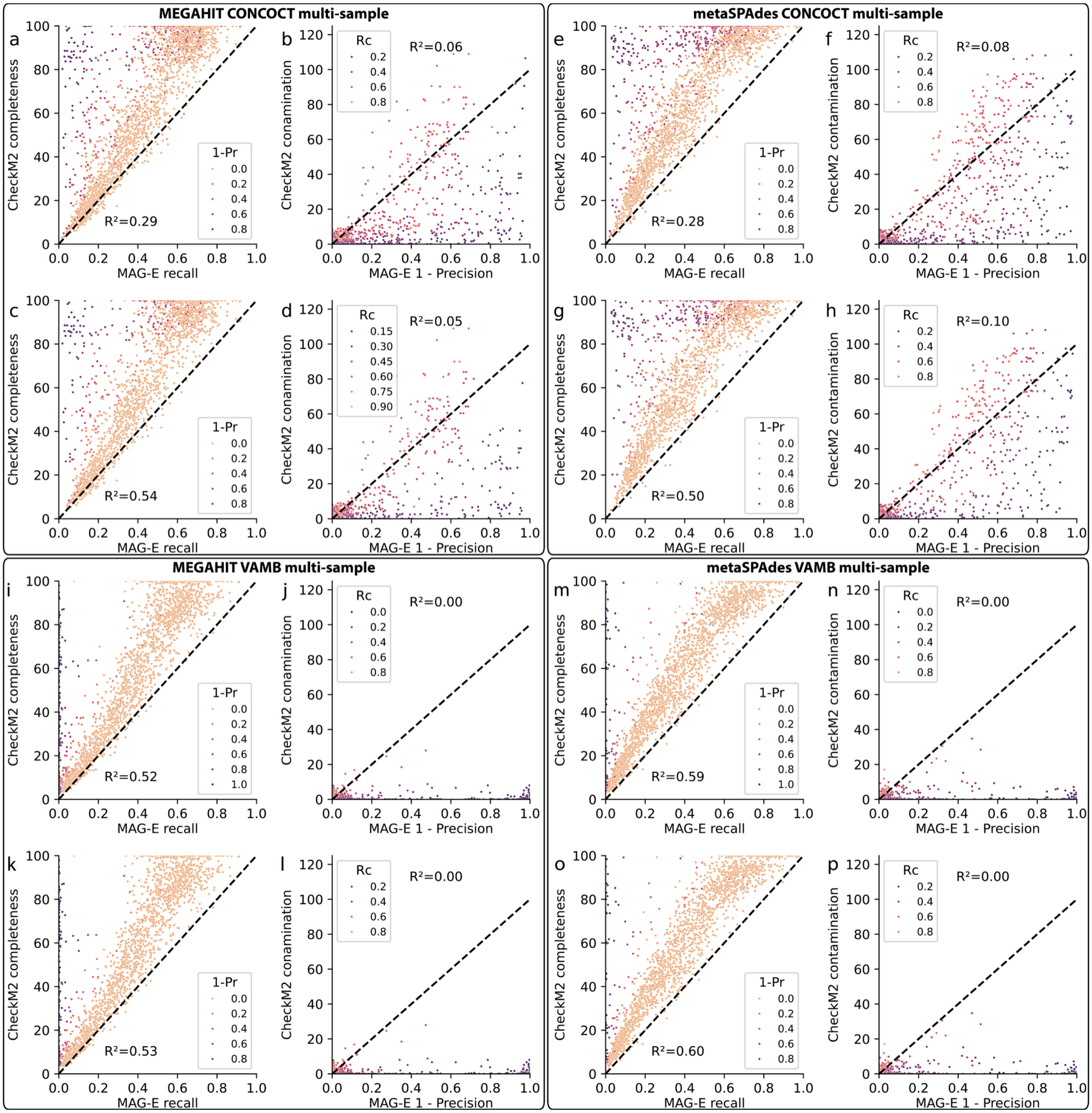

Supplementary Figure 11 | Evaluation of CheckM2 completeness and contamination versus ground-truth, per-genome MAG-E evaluation for CONCOCT and VAMB in multi-sample mode. Same as **Fig. S7** for multi-sample mode.

bioRxiv preprint doi: https://doi.org/10.64898/2026.04.06.712906; this version posted April 9, 2026. The copyright holder for this preprint (which was not certified by peer review) is the author/funder, who has granted bioRxiv a license to display the preprint in perpetuity. It is made available under aCC-BY 4.0 International license.

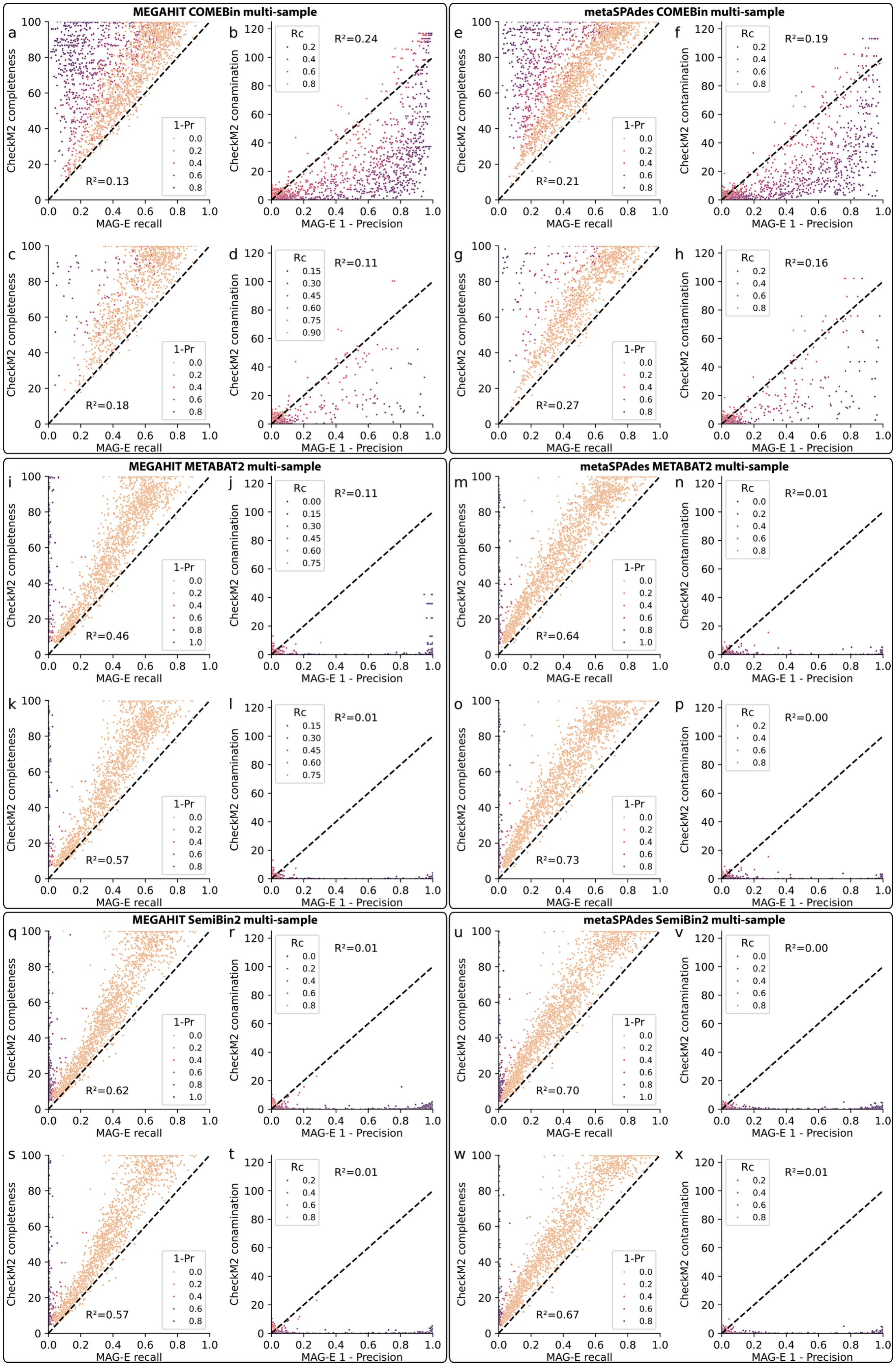

Supplementary Figure 12 | Evaluation of CheckM2 completeness and contamination versus ground-truth, per-genome MAG-E evaluation for COMEBin, METABAT2, and SemiBin2 in multi-sample mode. Same as **Fig. S8** for multi-sample mode.

bioRxiv preprint doi: https://doi.org/10.64898/2026.04.06.712906; this version posted April 9, 2026. The copyright holder for this preprint (which was not certified by peer review) is the author/funder, who has granted bioRxiv a license to display the preprint in perpetuity. It is made available under aCC-BY 4.0 International license.

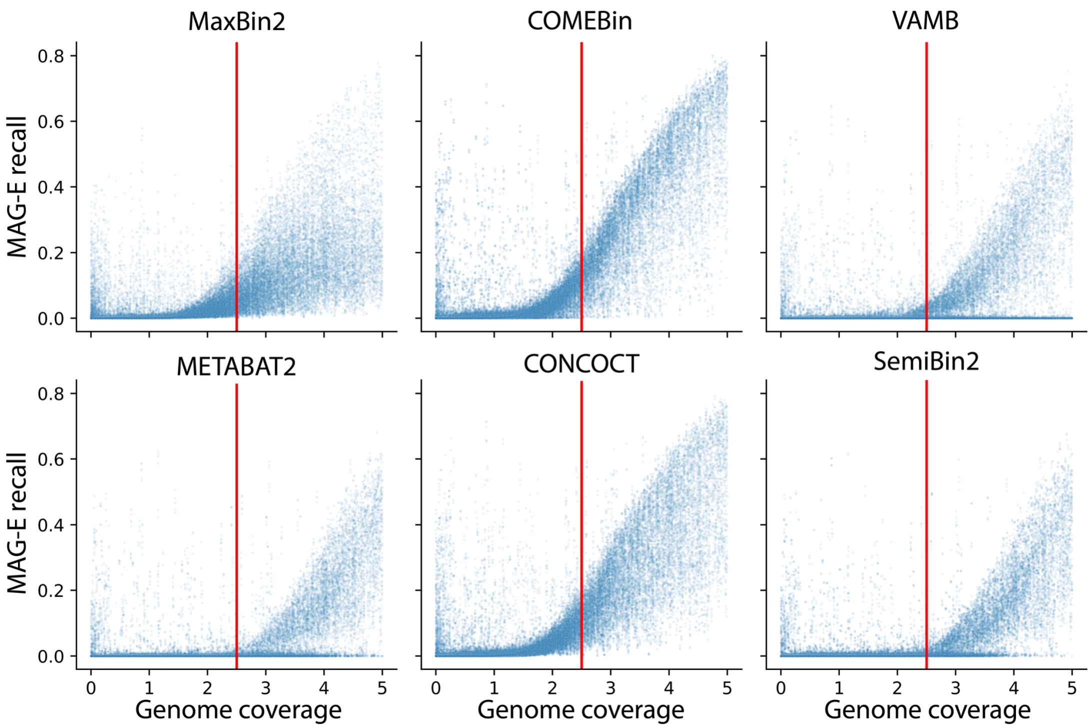

Supplementary Figure 13 | Recall (y-axis) vs. expected coverage (x-axis) for all ground-truth genomes across the different MAG-pipelines, stratified by each binner, demonstrating a substantial increase after 2.5x coverage (red lines).

bioRxiv preprint doi: https://doi.org/10.64898/2026.04.06.712906; this version posted April 9, 2026. The copyright holder for this preprint (which was not certified by peer review) is the author/funder, who has granted bioRxiv a license to display the preprint in perpetuity. It is made available under aCC-BY 4.0 International license.

Supplementary tables

Supplementary Table 1 | Summary of past binning benchmark studies. Listed for each study is: whether the metagenomic datasets considered of simulated or real sequencing reads; whether the exact genomic sequence that the MAGs derive from was known, i.e., whether a ground truth was used; dataset complexity, describing the microbiome source or number of elements in the dataset; number of samples in the dataset; whether the analysis examined the binning of contigs with particular properties; evaluated tools used to quantify MAG quality; and compared the performance of different short-read metagenomic assemblers, binning modes, and binners in combination, rather than just the performance of binning.

Study (dataset)	Simulated or real	Ground truth	Dataset complexity	Dataset size (samples)	Contig-level	Quality filtering	MAG pipeline
Sczyrba et al., 2017[21] (high complexity)	Simulated	Yes	596 genomes, 478 circular elements	1	No	No	No
Sczyrba et al., 2017[21] (medium complexity)	Simulated	Yes	132 genomes, 100 circular elements	1	No	No	No
Sczyrba et al., 2017[21] (low complexity)	Simulated	Yes	40 genomes, 20 circular elements	1	No	No	No
Meyer et al., 2022[22] (marine)	Simulated	Yes	77 genomes, 200 circular elements	10	No	No	No
Meyer et al., 2022[22] (plant)	Simulated	Yes	496 genomes, 398 circular elements	21	No	No	No
Meyer et al., 2022[22] (strain madness)	Simulated	Yes	408 genomes	100	No	No	No
Maguire et al., 2020[24]	Simulated	Yes	30 genomes, 65 plasmids	1	Yes	Yes	No
Nelson et al., 2020[19] (mock)	Real	Yes	20 genome mock community	1	Yes	No	No
Nelson et al., 2020[19] (tara oceans)	Real	Partially	Marine microbiome	1293	Yes	No	No
Mattock et al., 2023[17]	Real	No	Cow rumen	42	Yes	Yes	No
Han et al., 2025[23]	Real	No	Marine, cheese, human, sludge	5	No	No	No
Yue et al., 2020[44] (chicken gut)	Real	No	Chicken gut	4	No	No	No
Yue et al., 2020[44] (simulated)	Simulated	Yes	CAMI 1 high, medium, low complexity	3	No	No	No

Supplementary Table 2. Recall, precision, and F-score of all isolate genomes and MAGs for each of the 36 pipelines, presented in **Fig. 1b**.

bioRxiv preprint doi: https://doi.org/10.64898/2026.04.06.712906; this version posted April 9, 2026. The copyright holder for this preprint (which was not certified by peer review) is the author/funder, who has granted bioRxiv a license to display the preprint in perpetuity. It is made available under aCC-BY 4.0 International license.

Supplementary Table 3. MAG-E recall, precision, and F-score of recoverable genomes across 100 samples for each of the 48 MAG pipelines, presented in **Figs. 2a,b, S2a, S3,** and **S5a-f**.

Supplementary Table 4. Marginal mean MAG-E recall estimated by linear mixed models (**Methods**) for each of the 48 MAG pipelines, presented in **Figs. 2c,e,g** and **3a,c**.

Supplementary Table 5. Marginal mean MAG-E precision estimated by linear mixed models (**Methods**) for each of the 48 MAG pipelines, presented in **Figs. 2d,f,h** and **3b,d**.

Supplementary Table 6. Marginal mean MAG-E F-score estimated by linear mixed models (**Methods**) for each of the 48 MAG pipelines, presented in **Figs. S2b-d and S6a,b.**

Supplementary Table 7. Contig-level recall of the all contigs, prophage elements, and shared elements of the recoverable genomes across 100 samples for METABAT2, SemiBin2 and COMEBin run in multi-sample mode following assembly by metaSPAdes, presented in **Fig. 4c**.

Supplementary Table 8. Contig-level recall of all contigs, prophage elements, and shared elements of the recoverable genomes across 100 samples for METABAT2 run in single-sample, partial multi-sample and multi-sample mode, presented in **Fig. 4d.**

Supplementary Table 9. Contig-level recall of all contigs, prophage elements, and shared elements of the recoverable genomes across 100 samples for SemiBin2 run in single-sample, partial multi-sample and multi-sample mode, presented in **Fig. 4e.**

Supplementary Table 10. Contig-level recall of all contigs, prophage elements, and shared elements of the recoverable genomes across 100 samples for COMEBin run in single-sample, partial multi-sample and multi-sample mode, presented in **Fig. 4f.**

Supplementary Table 11. Recall, precision, and F-score of the recoverable genomes from each of 30 pipelines classified into MIMAG categories by CheckM2, with and without GUNC filtering, presented in **Fig. 5a,b,e,f.**

Supplementary Table 12. Recall, precision, F-score, CheckM2 completeness and CheckM2 contamination for all genomes ≥ 2.5x coverage that were successfully binned, presented in **Figs. 5c,d,g,h, S7-12**.

Supplementary Table 13. List of 100 metagenomic samples used for evaluation of MAG pipelines in this work.
